# Intensive care unit nurses’ knowledge, attitudes and practices of COVID-19 infection prevention and control

**DOI:** 10.4102/sajid.v38i1.478

**Published:** 2023-06-29

**Authors:** Onga Bangani, René English, Angela Dramowski

**Affiliations:** 1 Division of Health Systems and Public Health, Department of Global Health, Faculty of Medicine and Health Sciences, Stellenbosch University, Cape Town, South Africa; 2 Department of Paediatrics and Child Health, Faculty of Medicine and Health Sciences, Stellenbosch University, Cape Town, South Africa

**Keywords:** COVID-19, infection prevention and control, IPC, intensive care unit, ICU, knowledge, attitudes, practices

## Abstract

**Background:**

Intensive care units (ICUs) had to rapidly adapt infection prevention and control (IPC) practices during the coronavirus disease 2019 (COVID-19) pandemic.

**Objectives:**

To determine ICU nurses’ COVID-19 IPC-related knowledge, attitudes, practices, and perceptions.

**Method:**

A mixed-methods study was conducted at the Groote Schuur Hospital ICU, Cape Town, South Africa (20 April 2021 and 30 May 2021). Participants completed anonymous, self-administered, knowledge, attitudes and practices (KAP) questionnaires. Individual interviews were conducted regarding nurses’ lived experiences and perceptions of COVID-19 IPC in critical care.

**Results:**

In total, 116 ICU nurses participated (93.5% response rate) including 57 professional nurses (49%), 34 enrolled nurses (29%) and 25 enrolled nursing assistants (22%); young females (31–49 years) predominating (*n* = 99; 85.3%). Nurses’ overall COVID-19 IPC knowledge scores were moderately good (78%); professional nurses had greater knowledge of COVID-19 transmission (*p* < 0.001). Intensive care unit nurses’ attitude scores towards COVID-19 IPC were low (55%), influenced by limited IPC training, insufficient time to implement IPC and shortages of personal protective equipment (PPE). Respondents’ scores for self-reported COVID-19 IPC practices were moderate (65%); highest compliance rates were for hand hygiene after touching patient surroundings (68%). Only 47% ICU nurses underwent N95 respirator fit-testing despite working in a COVID-19 ICU.

**Conclusion:**

Regular COVID-19 IPC training is needed to equip ICU nurses with the knowledge and skills to prevent healthcare-associated COVID-19 transmission. Enhanced IPC training and consistent PPE availability may support more favourable attitudes and better IPC practices. Comprehensive IPC and occupational health support should be offered to ensure ICU nurses’ wellbeing during pandemics.

**Contribution:**

Enhanced IPC training and consistent PPE availability may support better attitudes and IPC practices.

## Introduction

Coronavirus disease 2019 (COVID-19) is a severe respiratory illness caused by the severe acute respiratory syndrome coronavirus 2 (SARS-CoV-2), a novel β-coronavirus emerged in Wuhan, China, in late 2019 and rapidly spread worldwide.^1^ COVID-19 is a highly infectious disease spread primarily through respiratory aerosols from human to human.^1^ Although most COVID-19 infections are mild or even asymptomatic, up to 5% of patients will require hospital care.^2^ Severe COVID-19-associated pneumonia is the leading indication for admission to the intensive care unit (ICU).^2^

As a result of the COVID-19 pandemic, the working conditions for ICU staff globally became increasingly difficult. The pandemic resulted in surges in ICU bed demand, higher patient-to-nurse ratios and increased staff absenteeism because of COVID-19 infection-related sick leave. Furthermore, ICU nurses were particularly at risk of developing mental health problems, including depression, anxiety and burnout during the pandemic.^3^

South African healthcare facilities follow the national Infection Prevention and Control (IPC) strategic framework published in March 2020 including those specifically developed for COVID-19 IPC in April 2020, which outline the recommended IPC measures for different clinical areas, clinical activities and staff.^4,5^ These guidelines are intended to provide direction for healthcare workers (HCWs) to ensure both occupational and patient safety in the care of patients with infectious diseases. Specific recommendations in the ICU setting include the use of the closed endotracheal tube and patient suctioning, bed spacing of 3 m and dedicated patient care equipment.^4^

Intensive care unit nurses’ understanding, acceptance and implementation of national and local COVID-19 IPC practice guidelines are key to ensuring safe ICU working environments, as they carry the primary responsibility for IPC policy implementation and practices.^6^ Acknowledging the key role of ICU nurses in attaining COVID-19 IPC compliance, several studies from low-and middle-income countries (LMICs) surveyed HCWs’ knowledge, attitudes and practices (KAP) during the COVID-19 pandemic.^1,7,8,9^ Studies from Uganda, Ethiopia and Nepal concluded that HCWs in LMIC settings had adequate knowledge and a positive attitude to IPC during the COVID-19 pandemic, but to our knowledge at the time of our study, none had included interviews with ICU nurses.^8,9,10,11^ Intensive care unit nurses are highly qualified, skilled and experienced in caring for acutely ill patients and represent a cadre of healthcare workers with high exposure to COVID-19 and high risk for suffering adverse work-related mental health effects during the pandemic.^12^ We aimed to describe ICU nurses’ KAP and lived experiences of COVID-19 IPC at a tertiary hospital, dedicated COVID-19 ICU in Cape Town, South Africa.

## Methods

### Study setting and population

This study was conducted in the COVID-19 dedicated ICUs at Groote Schuur Hospital (GSH), Cape Town, South Africa. Groote Schuur Hospital is a 991-bed academic hospital situated in the City of Cape Town Metropolitan health district, which was the epicentre of COVID-19 infection in the Western Cape province during the first, second and third infection waves.^13^ Groote Schuur Hospital has 10 ICUs (with six beds each and one isolation unit with seven beds), of which seven were designated for COVID-19 patients (total bed capacity 43) since 05 April 2020. The seven COVID-19 ICUs were staffed by 120 permanent nurses and four sessional nurses. Every nurse was responsible for implementing IPC in the ICU. The nursing categories consisted of professional nurses (PN), enrolled nurses (EN) and enrolled nursing assistants (ENA).^14^ Professional nurses are senior nurses with a 4-year nursing degree who supervise EN and ENA, perform skilled nursing procedures, administration of patient medication as well as administrative duties. Each PN was responsible for the care of two ventilated patients, executing doctors’ orders and administering intravenous infusion medication. Enrolled nurses have a 2-year nursing diploma programme and cared for non-ventilated COVID-19 patients and administered oral medications only. Enrolled nursing assistants have a 1-year diploma and provide supportive duties for the PN and ENA, personal hygiene care for patients and assisted with environmental cleaning in the ICU.

Groote Schuur Hospital has an on-site Unit for IPC employing three IPC nurse specialists and one nursing clinical facilitator. Prior to the COVID-19 pandemic, the ICU clinical facilitator conducted in-service training every two weeks for ICU nurses. From the onset of the COVID-19 pandemic, a single face-to-face COVID-19 IPC in-service training session was held for each ICU, and nurses were encouraged to watch a COVID-19 IPC video on the hospital computers. The limited training time for COVID-19 IPC resulted from the huge pressure of COVID-19 patient admissions to the ICU, which precluded ICU nurses from attending more in-service training sessions.

All patients admitted to the COVID-19 ICUs had nasopharyngeal swabs taken to confirm the presence of SARS-CoV-2 using reverse transcription polymerase chain reaction (RT-PCR) tests. The Occupational Health Service at GSH monitored HCWs’ COVID-19 infection rates at the facility from April 2020 onwards.

### Study design

This research was designed as a mixed-methods study, with simultaneous data collection for both study components between 20 April 2021 and 30 May 2021.^11^ The questionnaire and interviews were used to explore ICU nurses’ knowledge, attitudes and practices related to COVID-19 IPC. Quantitative data were collected using a self-administered anonymous, 33-item questionnaire to assess participants’ KAP regarding COVID-19 IPC, as well as participants’ demographics (nursing category, work experience and risk factors for severe COVID-19 disease). We developed a semi-structured interview questionnaire comprising six open-ended questions to further explore and provide insights into the three quantitative constructs covered in the quantitative assessment (see Appendix 1).

### Sampling strategy

A convenience sampling strategy was employed, enrolling PN, EN and ENA staff categories from the COVID-19 ICUs. The principal investigator (PI) visited all seven COVID-19 dedicated ICUs five times during the study period (including day and night shifts) to explain the purpose of the study and to invite all the nurses working in the COVID-19 ICU to participate. Nurses completed the questionnaires manually, in English, providing written informed consent, and the questionnaires were collected after each 12-h shift by the study PI.

Purposive sampling was used to select participants to interview for the qualitative arm of this study.^15^ The sample included all the nursing categories (PN, EN and ENA) working in COVID-19 ICUs with varying years of work experience, gender (female and male), and education level (diploma and degree). The participants included staff from the day and night shifts. The purpose of the study was explained to each participant. Interviews were conducted in English in a private room. The data were transcribed, analysed, coded and triangulated with the quantitative data.

### Measurement instruments

#### Self-administered questionnaire

A survey instrument was designed based on previous KAP studies of healthcare-associated infection.^10^ The instrument was adapted and modified to assess the ICU nurses’ KAP of COVID-19 IPC. The closed-ended questionnaire consisted of four parts: participant demographics (10 questions), knowledge (9 questions), attitudes (15 questions) and practices (8 questions). Scoring criteria were set, giving a score of ‘1’ for a correct answer and a score of ‘0’ for an incorrect answer. Knowledge questions assessed nurses’ understanding of COVID-19 IPC measures, attitude questions sought to determine the ICU nurses’ attitudes level towards COVID-19 IPC measures, while practice questions collected nurses’ self-reported practices regarding prevention of COVID-19 infection (see Appendix 2). The assessment of the correlation relationship between variables of knowledge, attitude and practice scores was conducted using Spearman’s rho correlation test. A pilot study was conducted to assess the validity and reliability of the questionnaire before using it. Two experts in infectious disease and public health evaluated the KAP questions. Pretesting of the instrument was done, and a questionnaire was completed by two operational managers and six ICU nurses who were excluded from the study sample.

#### Semi-structured qualitative question guide

The semi-structured qualitative guide was designed to complement the quantitative questionnaire and focused on exploring the lived experiences of COVID-19 IPC at the GSH dedicated COVID-19 ICUs. The semi-structured guide comprised six open-ended questions centred on experience in ICU, application of IPC measures during the COVID-19 pandemic, adaptation to new COVID-19 IPC guidelines, COVID-19 IPC training, COVID-19 precautions and how ICU nurses were coping in their daily practice and personal lives.

### Data management and statistical analysis

Quantitative data from the manually completed questionnaires were entered into a RedCAP database by the PI and analyses were performed using Stata^®^ Statistical software version 15.0 (Stata Corp, College Station, Texas, United States). The categorical data were summarised using the frequency and percentages. The proportions for KAP were compared by nursing job category (EN vs ENA vs PN). The chi-square or Fisher’s exact test was used for comparing categorical data. The Spearman’s rho correlation test was used to find the relationship between knowledge, attitude and practices, following the conversion of individual items in the questionnaire to overall percentages of the correct answers (scoring 1 for correct and 0 for incorrect responses). The statistically significant level was set in all tests at *p* < 0.05.

### Qualitative analysis

Qualitative data were obtained by posing six pre-specified open-ended questions to participants. Participants’ responses were audio-recorded and transcribed verbatim, and a six-step thematic analysis approach was employed: familiarisation with data, generating initial codes, searching for themes, reviewing themes, defining, naming themes and producing a report.^16^ Emerging themes developed from respondents’ lived experiences while working with COVID-19 IPC precautions in the ICU, and direct participants’ quotes that best reflected these emerging themes were selected.

### Mixed methods integration

The interconnected themes from the qualitative data were integrated and aligned with the results of the quantitative data.^17^ Themes that represented the knowledge, attitudes and practices of ICU nurses regarding COVID-19 IPC were identified, and conclusions were drawn in conjunction with the quantitative data.

### Ethical considerations

Ethics approval was obtained from the Human Health Research Ethics Committee of the University of Cape Town (774/2020). All participants provided written informed consent.

### Results

#### Participant demographics

Of 124 eligible COVID-19 ICU nurses, 116 nurses participated in the study (response rate of 93.5%). Respondents were predominantly young females (*n* = 99; 85.3%; 59.5% aged 31–49 years) (Table 1). Respondents comprised the following nursing categories: PN (*n* = 57; 50.0%), EN (*n* = 34; 29.3%) and ENA (*n* = 25; 22.0%). Almost half of the respondents had substantial (> 10 years) ICU nursing experience (*n* = 51, 45.9%). Approximately one-quarter of all nurses had one or more underlying medical conditions that placed them at increased risk for severe COVID-19 disease (*n* = 32, 28%). Fifty-two nurses (44.8%) reported previously having undergone one or more COVID-19 RT-PCR tests, and 21 (18.1%) were previously diagnosed with COVID-19 infection at the time of the study.

**TABLE 1:** Participant demographic characteristics (*N* = 116).

Variable	*N*	%
**Gender**		
Female	99	85.3
Male	17	14.7
**Age range (in years)**		
18–30	17	15.0
31–49	69	60.0
50+	30	25.0
**Work experience (years)**		
< 1	8	7.0
1–5	28	24.5
5–10	27	23.7
> 10	51	44.8
**Nursing job category**		
Professional nurse	57	49.0
Enrolled nurse	34	29.0
Enrolled nursing assistant	25	22.0
Nurses with underlying medical conditions	32	28.0
**Risk factors for severe COVID-19 infection:**		
Diabetes	9	7.8
Hypertension	16	13.8
Heart disease	1	2.6
Chronic lung disease	3	0.9
Previous pulmonary tuberculosis	3	2.6
Living in a household with people vulnerable to severe COVID-19 disease	27	23.3
Previously underwent a COVID RT-PCR testing	52	44.8
Previously diagnosed with COVID-19 infection	21	18.1
Required ≥ 1 quarantine periods after high-risk COVID-19 exposure in the ICU	27	23.3

COVID-19, coronavirus disease 2019; RT-PCR, reverse transcription polymerase chain reaction; ICU, intensive care unit.

#### Intensive care unit nurses’ knowledge of COVID-19 infection prevention and control

Respondents’ overall knowledge scores pertaining to COVID-19 transmission mechanisms and prevention measures in the ICU were moderate to good (78%). Professional nurses had significantly higher scores than other nursing categories regarding the role of aerosol transmission of the SARS-CoV-2 virus (*p* < 0.001, Table 2). Intensive care unit nurses had good knowledge (82%) regarding the importance of hand hygiene to remove COVID-19 viral particles from the skin; 78% of respondents believed that alcohol hand rub is as effective for decontaminating hands possibly contaminated with COVID-19, as handwashing with soap and water. Intensive care unit nurses had good knowledge (91%) of the indications for terminal cleaning and disinfection of hospital rooms. Most (84%) respondents were aware that all patients admitted to ICU were tested for COVID-19 infection.

**TABLE 2:** Intensive care unit nurses’ knowledge regarding infection prevention and control precautions for COVID-19.

Knowledge questions (correct response)	Total (*N* = 116)		Professional nurse (*n* = 57)		Enrolled nurse (*n* = 34)		Enrolled nursing assistant (*n* = 25)		*p*
	*n*	%	*n*	%	*n*	%	*n*	%	
The hospital environment (surfaces and water) is the main source of COVID-19 transmission to staff (false)	65	56.0	35	61.4	19	56.0	11	44.0	< 0.001
Handwashing with soap and water is sufficient to remove COVID-19 viral particles from the skin (true)	97	81.8	47	82.4	28	82.3	20	80.0	0.654
Alcohol hand rub is as good as handwashing with soap and water for decontaminating hands that are possibly contaminated with COVID-19 (true)	91	78.4	45	78.9	28	82.3	18	72.0	0.520
Hand hygiene should be done before putting on a mask or N95 respirator (true)	110	94.8	55	96.4	32	94.1	23	92.0	0.839
Patients with COVID-19 infection require the same infection control precautions as patients infected with antibiotic-resistant bacteria (false)	78	67.2	40	70.2	22	64.7	16	64.0	0.864
Terminal cleaning and disinfection of the hospital room is required following the discharge of patients with COVID-19 (true)	106	91.2	52	91.2	31	91.1	23	92.0	0.305
COVID-19 infection is spread mainly by the respiratory route (true)	102	87.9	50	87.7	28	82.3	20	80.0	0.664
Open suctioning and intubations are aerosol-generating procedures (true)	105	90.5	54	94.7	28	82.3	23	92.0	0.999
The GSH Unit for Infection Prevention and Control performs active surveillance by testing every patient admitted to ICU for COVID-19 (true)	98	84.4	49	85.9	27	79.4	22	88.0	0.999

*Source:* Adapted from Dramowski A, Whitelaw A, Cotton MF, et al. Healthcare-associated infections in children: knowledge, attitudes and practice of paediatric healthcare providers at Tygerberg Hospital, Cape Town. Paediatrics and international child health. 2016 Jul 2;36(3):225–231.

GSH, Groote Schuur Hospital; ICU, intensive care unit.

#### Intensive care unit nurses’ attitudes to COVID-19 infection prevention and control

Intensive care unit nurses’ attitudinal scores with regard to COVID-19 IPC were marginally positive (55%), (Table 3). Half of the respondents (51%) reported that ICU nurses had a generally positive attitude towards COVID-19 IPC. However, half of the respondents (50%) believed that they had received insufficient training in COVID-19 IPC measures before admitting infected patients to the ICU. Many respondents (72%), especially PNs (88%), (*p* < 0.005) believed that the high workload during the COVID-19 pandemic resulted in decreased time to adhere to standard IPC practices in the ICU. Professional nurses were more likely to agree with the statement that there was adequate availability of PPE in the ICU, compared to EN and ENA (70.2% vs 56% and 52%; *p* = 0.048).

**TABLE 3:** Intensive care unit nurses’ attitudes regarding infection prevention and control precautions for COVID-19.

Attitude questions (desired response)	Total (*N* = 116)		Professional nurse (*n* = 57)		Enrolled nurse (*n* = 34)		Enrolled nursing assistant (*n* = 25)		*p*
	*n*	%	*n*	%	*n*	%	*n*	%	
I received adequate teaching about IPC during undergraduate and in-service training (agree)	57	49.1	30	53.3	16	47.1	11	44.0	0.489
I received sufficient training in IPC for COVID-19 before we began admitting infected patients to the ICU (agree)	70	60.3	39	68.3	20	59.1	11	44.0	0.008
I received sufficient training in the procedures for safely donning and doffing PPE for COVID-19 (agree)	58	50.0	31	54.4	16	47.0	11	44.0	0.520
GSH ICU has adequate isolation facilities to reduce potential infection transmission of COVID-19 (agree)	57	49.1	28	49.1	16	47.0	13	52.0	0.361
Prevention of in-hospital COVID-19 staff and patient infections is the responsibility of the IPC staff (disagree)	52	44.8	24	42.1	18	52.9	10	40.0	0.875
Staff members who do not perform hand hygiene or ignore infection control recommendations should be reprimanded (agree)	58	50.0	29	50.9	17	50.0	12	48.0	0.240
The ICU nursing staff have a positive attitude towards COVID-19 IPC (agree)	59	51.1	30	53.0	16	47.0	13	52.0	0.217
Transmission of COVID-19 infection can be prevented by taking antibiotics (disagree)	67	58.1	37	65.1	19	56.0	11	44.0	0.218
The information and training about IPC for COVID-19 provided to ICU nursing staff was adequate to empower staff to protect themselves (agree)	58	50.0	29	51.0	17	50.0	12	48.0	0.228
The high workload during the COVID-19 pandemic resulted in decreased time to adhere to standard IPC practices in the ICU (agree)	84	72.4	50	88.1	20	59.0	14	56.0	0.048
I am familiar with the COVID-19 IPC guidelines for the ICU (agree)	61	62.9	31	54.3	17	50.0	13	52.0	0.322
During the COVID-19 pandemic, there has been adequate availability of PPE in ICU (agree)	72	66.4	40	70.2	19	56.1	13	52.0	0.048
During the COVID-19 pandemic, there has been adequate availability of cleaning supplies and disinfectants in the ICU (agree)	66	52.6	34	59.6	19	52.9	13	52.0	0.195
The correct PPE to care for COVID-19 patients is always available in the ICU (agree)	68	59.0	36	63.1	18	53.1	14	56.0	0.474

*Source:* Adapted from Dramowski A, Whitelaw A, Cotton MF, et al. Healthcare-associated infections in children: knowledge, attitudes and practice of paediatric healthcare providers at Tygerberg Hospital, Cape Town. Paediatrics and international child health. 2016 Jul 2;36(3):225–231.

PPE, personal protective equipment; IPC, infection prevention and control; ICU, intensive care unit; GSH, Groote Schuur Hospital.

#### Intensive care unit nurses’ practices for COVID-19 infection prevention and control

Respondents’ overall self-reported practice scores were moderate (65%). The highest reported compliance was for the performance of hand hygiene after touching the patient’s surroundings (68%), as well as the performance of hand hygiene before and after touching COVID-19-infected patients (67%). Less than half of respondents (47%), especially ENAs (40%), *p* = 0.039 (Table 4), had undergone N95 respirator fit-testing, despite working in a high-risk environment for COVID-19 transmission. One-third of staff (30%) reported for duty despite having symptoms of possible COVID-19 infection. Self-reported adherence to protective measures was low to moderate with only 66% wearing all recommended PPE when nursing COVID-19-infected patients. Only 56% reported adhering to transmission-based precaution signs in the ICU. Intensive care unit nurses reported low uptake rates for the annual influenza vaccination in 2019–2020 (46%).

**TABLE 4:** Intensive care unit nurses’ practices regarding infection prevention and control precautions for COVID-19.

Practice questions (desired response)	Total (*N* = 116)		Professional nurse (*n* = 57)		Enrolled nurse (*n* = 34)		Enrolled nursing assistant (*n* = 25)		*p*
	*n*	%	*n*	%	*n*	%	*n*	%	
I always wear the correct PPE when dealing with COVID-19-infected patients (agree)	77	66.4	39	68.4	20	59.2	18	52.0	0.662
I always recognise and adhere to the transmission-based precaution signs in ICU (agree)	69	59.4	37	65.1	21	62.0	15	60.0	0.698
I have performed fit-testing for use of N95 respirators (agree)	55	47.4	30	53.0	15	44.1	10	40.0	0.039
When I am sick, I feel obliged to come to work because we are short staffed (disagree)	61	53.1	33	58.1	18	53.0	10	40.0	0.687
All staff in the ICU should receive an annual influenza vaccine (agree)	64	55.2	34	60.0	18	53.0	12	48.0	0.979
Staff who develop possible symptoms of COVID-19 should not report for duty (agree)	71	61.0	39	65.1	20	59.0	14	56.0	0.772
I perform hand hygiene before and after touching patients with COVID-19 infection (agree)	78	67.2	41	71.2	22	63.1	15	60.0	0.772
I perform hand hygiene after touching the patient’s surroundings like beds, tables, doors, etc. (agree)	79	68.1	40	70.1	23	68.1	16	64.0	0.858

*Source:* Adapted from Dramowski A, Whitelaw A, Cotton MF, et al. Healthcare-associated infections in children: knowledge, attitudes and practice of paediatric healthcare providers at Tygerberg Hospital, Cape Town. Paediatrics and international child health. 2016 Jul 2;36(3):225–231.

PPE, personal protective equipment; ICU, intensive care unit.

#### Correlation between COVID-19 infection prevention and control knowledge, attitudes and practices

Table 5 shows the relationship between COVID-19 IPC KAP. There was a significant positive correlation (*r* = 0.484, *p* = 0.047) between higher knowledge scores and desired practices. There was also a significant positive correlation between positive attitudes and desired practices (*r* = 0.511, *p* < 0.001). However, the knowledge and attitudes scores were not significantly correlated (*r* = 0.207, *p* = 0.407). Thus, knowledge appears not to influence attitudes but does influence practices. Attitudes appear to influence practices.

**TABLE 5:** Correlation between knowledge, attitudes and practice scores.

Variables	Correlation coefficient	*p*
Knowledge – attitudes	0.207	0.407
Knowledge – practices	0.484	0.047
Attitudes – practices	0.511	< 0.001

Note: Correlation is significant at *p* < 0.05.

### Qualitative themes

In the qualitative arm of the study, 11 participants were recruited and were predominantly females (*n* = 7, 64%), aged 31–49 years (60%), including five PNs, three ENs and three ENAs. Half (50%) of the participants had over 10 years of experience in ICU nursing. Three interconnected themes comprising coping mechanisms and interventions to limit the occupational risk of COVID-19 infection emerged: inadequate IPC training during the COVID-19 pandemic, limited adherence to COVID-19 IPC practices, and emergence of coping methods, resilience and teamwork among ICU nurses during the COVID-19 pandemic.

#### Theme 1. Inadequate infection prevention and control training during the COVID-19 pandemic

A key theme that emerged from the qualitative data was that IPC training during the COVID-19 pandemic was considered inadequate for ICU nurses’ needs. Many expressed frustrations at the lack of training on COVID-19 IPC and the intermittent availability of PPE. Respondents also highlighted that rapid changes to protocols and procedures were needed and rapidly communicated to them during the pandemic as new knowledge emerged, to keep abreast of current COVID-19 IPC best practices. Respondents demanded regular training on COVID-19 IPC to ensure compliance:

‘COVID-19 is a new disease and has changed the critical environment completely. New information continues to surface regarding preventative measures. We need regular in-services training on COVID-19 IPC, please’. (Responder 09, male, ENA)

‘I need a continuous update on new evidence regarding COVID-19 IPC … on how to conduct infection prevention controls in the ICU environment and most importantly how to prevent ourselves from contracting this disease’. (Responder 11, female, PN)

#### Theme 2. Limited adherence to COVID-19 infection prevention and control practices

Respondents understood that the COVID-19 IPC guidelines were intended to combat virus transmission to HCWs and patients. Respondents commonly described how they applied COVID-19 IPC precautions to protect themselves from infection but had limited time to implement these IPC measures despite their intent to comply. The increased workload because of increased responsibilities, the volume of patients and the complexity of caring for COVID-19 patients were further factors that negatively influenced their compliance:

‘We tried [*to*] comply with COVID-19 IPC all the time, but it is difficult and sometimes you forget because we found ourselves looking after more than three intensive patients, ventilated on inotropic support, and besides that, you have guided our colleague from other departments, who have no ICU experience’. (Responder 05, female, PN)

‘The workload is too much, and we have minimal time to follow the COVID-19 IPC due to the severe illness of our patients, but we try all the time, but COVID patients are complicated’. (Responder 09, male, ENA)

‘It is very strenuous working with COVID patients because patients are critically ill all the time. It is impossible to adhere to the IPC protocol for the latter because we are trying to save lives. We acknowledge the importance of the COVID-19 IPC, we have limited time’. (Responder 06, female, EN)

#### Theme 3. Emergence of coping methods, resilience and teamwork among intensive care unit nurses during the COVID-19 pandemic

During the pandemic, ICU nurses had to balance the need to protect themselves and their families, while doing their best to ensure optimal patient outcomes under difficult circumstances at work and in their personal lives. Nurses expressed the strongest emotions regarding their care of patients with COVID-19 pneumonia or acute respiratory distress syndrome (ARDS) and expressed that they felt the weight of the responsibility of caring for these patients:

‘COVID-19 patients are very infectious. I feel vulnerable to contracting COVID-19 infection and spreading to my family. It also feels like everything is weighing heavy on us during this COVID-19 pandemic’. (Responder 07, female, EN)

Respondents appeared to have changed their behaviours within the workplace and home and adopted a range of coping strategies such as leaving their work clothes in the garage, not hugging their families, praying, exercising and other self-care measures:

‘When working in the COVID ICU, we changed our clothes before and after the shift. We put on PPE, aprons and N95 masks. After the shift, we changed back to nursing uniforms. When arriving home, I leave my working clothes, shoes, and bag in the garage, take a full shower, and do hand hygiene. We are trying to protect my children and the rest of my family from COVID-19 infection’. (Responder 08, male, PN)

The high workload during the pandemic and the low patient survival rate diminished the morale of ICU nurses, and at times they became pessimistic about the care they provided to COVID-19 patients. The respondents were overwhelmed by the shortage of PPE and constantly changing IPC protocols and procedures. This negatively impacted their attitudes to and compliance with IPC measures. Yet, the ICU nurses shared sentiments like ‘we will survive’ and ‘we are determined to serve our patients.’ Intensive care unit nurses displayed resilience and coping mechanisms with the high workload of COVID-19 patients. Despite all the uncertainty, they remain committed to their frontline duties, returning to work each day to provide nursing care during the public health pandemic and displaying a high level of altruism and dedication, often at the expense of their own health:

‘It’s very stressful to work with COVID patients and the first precaution is to counsel yourself and be calm within, because if by any chance you are stressing, then you will miss out on a lot of things. So, as long as you are calm, I think that’s the first thing to protect yourself because you know what to do. Try by all means to adhere to IPC protocols’. (Responder 03, male, PN)

‘Yoh, it was so draining, draining … You feel exhausted. You feel like you must go off sick. Then you think if you go off sick, who is going to look after the patients. You must force yourself to go to work. Although you get exhausted and tired, you must force yourself because you pledged’. (Responder 10, female, RN)

Intensive care unit nurses also highlighted the importance of teamwork in caring for COVID-19 patients:

‘The workload has increased significantly in the ICU since COVID19 came. We need each other, teamwork was the key to navigating away through COVID-19 waves. COVID-19 patients are very heavy and you will need help from your colleague, that is why teamwork is very crucial’. (Responder 07, female, RN)

‘Generally, ICU work needs teamwork among nurses. COVID-19 patients are difficult to nurse because of their infectious status. Teamwork is required throughout the day. We are helping each other in these difficult circumstances’. (Responder 10, female, RN)

## Discussion

A mixed-methods design was used to assess the COVID-19 IPC KAP of ICU nurses working at an academic hospital in a middle-income setting. Most respondents had moderate-to-good knowledge levels related to COVID-19 IPC, similar to recent reports from Nigeria and South Africa.^18,19^ Knowledge scores were generally lowest among nurses with the least training (EN, ENA). Despite minimal COVID-19-specific training opportunities, ICU nurses’ Knowledge scores were moderate, possibly helped by staff members’ existing knowledge of IPC transmission precautions and extensive media coverage of COVID-19 preventive measures.

Qualitative responses, however, did not support this interpretation. The respondents commonly identified the lack of regular IPC training during the COVID-19 pandemic as a major setback. They expressed their concerns more broadly about a ‘new’ and ‘highly’ ‘infectious disease’, which in their opinion required IPC training for adequate patient management and safety. Furthermore, guidance and regular in-service training for COVID-19 IPC are needed to correct some misconceptions and empower all nursing categories with accurate scientific information.

Knowledge levels on the key concepts of COVID-19 IPC measures, such as hand hygiene, routes of infection transmission and environmental cleaning were good. Most respondents correctly understood that alcohol hand rub is an adequate measure to limit the direct and indirect contact spread of COVID-19 infection, with many describing hand hygiene as a ‘key’ mitigation measure. This is reassuring because a good knowledge of COVID-19 transmission patterns and prevention measures will improve ICU nurses’ practices and thereby limit occupational risk. However, the respondents expressed concern about their working environment, especially the rapid changes in protocols and the severity of illness among their patients. These results are in keeping with the sentiments expressed in previous reports of COVID-19 IPC in ICUs.^10,17^ The attitude of ICU nurses may affect their practice of COVID-19 infection prevention and control measures. In this study, we assessed the acceptance and experience of the COVID-19 IPC by ICU nurses. Most respondents demonstrated a marginally positive attitude towards COVID-19 IPC adherence and practice in the ICU. Our qualitative data suggest that attitudes are influenced by fear of infection, high workload and perceived negative impacts of COVID-19 on HCWs’ health and that of their families. Similar findings were observed in Uganda and Nigeria.^8,18^ The marginally positive attitude could possibly be attributed to the limited training in IPC, insufficient time to implement IPC protocols and PPE shortages. Respondents also reflected on the ICU environment and the complexity of patient management brought on by COVID-19 ARDS patients, as well as that introduced by new or frequently updated IPC measures.

Half of the respondents believed they had received inadequate training in procedures for safely donning and doffing PPE for COVID-19. Most respondents, however, felt that the high workload during the COVID-19 pandemic resulted in decreased time to adhere to standard IPC practices in the ICU. Respondents expressed similar concerns about the inconsistent availability of PPE and fears of contracting COVID-19 themselves. The availability of PPE in ICUs in protecting staff from COVID-19 infection and an adequate supply of PPE may therefore improve ICU nurses’ attitudes to work. The major demand caused limited availability of PPE, but the situation was corrected over time and may no longer be a major problem within this hospital.^20^

We found a positive relationship between positive attitudes and practices suggesting that attitude influences the respondents’ COVID-19 IPC practice. The self-reported compliance of ICU nurses with recommended COVID-19 IPC practice was moderate. Similar findings were reported from China regarding moderate to good compliance with COVID-19 IPC.^7^ Less than half of respondents had performed N95 respirator fit testing despite their high-risk work environment. Similar results were reported in a recent study from Nigeria.^21^ During the COVID-19 pandemic, qualitative fit testing was discontinued in Western Cape facilities (owing to the risk of cross-infection), and this may have contributed to the low numbers of staff who had been fit-tested.

Most respondents did not adhere to transmission-based precaution signs in the COVID-19 ICU. Factors for this low adherence did not emerge during the qualitative inquiry. The practice of effective preventative measures such as handwashing and donning of appropriate personal protective equipment (PPE) will ensure the safety of HCWs by reducing healthcare-associated COVID-19 transmission.

Qualitative data suggest that respondents were willing to adapt to rapid changes in COVID-19 IPC guidelines despite some having indicated that this added to their workload, which in turn had likely adversely affected their compliance with COVID-19 IPC recommendations. There was generally a positive attitude among the respondents towards COVID-19 IPC, and the respondents expressed a desire for more regular in-service training on new evidence-based COVID-19 IPC precautions. The infrastructure and shortage of nursing staff are long-standing issues, and the COVID-19 pandemic has compounded them. Their resolution requires interventions at an institutional level.^22^

We found low attitudes and some non-compliant IPC practices in these ICU nurses with regard to the management of COVID-19. In planning for future pandemics, the provision of an uninterrupted supply of PPE, ongoing IPC training opportunities and timeous and clear communication of relevant information may assist in encouraging IPC compliance and more positive attitudes. Given the risk of staff burnout during health crises, employee wellness and support should remain a priority for healthcare employers and managers.

The study’s major limitation is using self-reported rather than directly observed COVID-19 IPC practices. Reporting bias may have been introduced by the subjective nature of the survey. This was also a single-centre study, which limits generalisability of the findings. This study was performed before the COVID-19 vaccination was readily available in South Africa, which may have increased ICU nurses’ adherence to IPC measures, in an effort to reduce their personal infection risk. The Johnson & Johnson COVID-19 vaccine initially rolled out to South African healthcare workers in February 2021, providing 72% protection against severe disease and death from COVID-19.^23^ Future studies should evaluate COVID-19 vaccine uptake among ICU nurses, given their reported low uptake of the annual influenza vaccine.

This study has several strengths. To the authors’ knowledge, this is the first mixed-methods assessment study performed in a resource-limited setting. The qualitative data gives meaning and explanation to the quantitative results. Data for this study were collected immediately after the second peak of the COVID-19 pandemic, the timing of which allowed for study participants to share fresh experiences with explicit examples. One of the strengths of this study is that there is unlikely to be selection bias, as the survey studied ICU nursing staff, and the majority responded.

## Conclusion

Despite very limited specific training, ICU nurses’ COVID-19 IPC knowledge was moderately good, whereas nurses’ attitude scores towards COVID-19 IPC were low (55%). Nurses attitudes to COVID-19 IPC were influenced by limited IPC training, insufficient time to implement IPC precautions, and shortages of PPE. Inadequate training in IPC, difficulty in implementing COVID-19 IPC guidance and development of resilience in ICU nurses emerged as major themes in the qualitative study. This study highlighted the need for enhanced support in IPC and occupational health and safety to preserve the well-being of ICU nurses during the COVID-19 and future pandemics

## Appendix 1

### Semi-structured qualitative question guide

1. Tell me about your experience working in the (ICU).
2. How do you apply infection prevention protocols in the COVID-19 ICU?
3. How have you adapted IPC practices to COVID-19?
4. Have you received training on COVID-19 IPC?
5. What precautions are you taking to prevent yourself and patients from contracting COVID-19 infection in the ICU?
6. How are you coping during this COVID-19 pandemic?
7. Is there anything else that you would like to share regarding COVID-19 IPC?

## Appendix 2

**TABLE 1-A2:** Please answer the following questions with a cross in the appropriate box (X).

Demographics of intensive care nurses		
**1. Gender**		
Male		
Female		
**2. Age, (in years)**		
18–29		
31–49		
50+		
**3. Nursing category**		
Professional Nurse (PN)		
Enrolled Nurses (EN)		
Enrolled Nursing Assistants (ENA)		
**4. Work Experience, (in years)**		
< 1		
1–5		
5–10		
> 10		
**5. Do you have any underlying health conditions? Put a cross in the box of all that apply**		
Diabetes		
Hypertension		
Heart disease		
Chronic lung disease		
Previous pulmonary tuberculosis		
Autoimmune disease		
HIV and/or AIDS		
None of the above		
Prefer not to say		
6. Are you living in a household with people vulnerable to COVID-19 infection?	Yes	No
7. Did you need to go into quarantine after high-risk COVID-19 exposures in the ICU?	Yes	No
8. Did you go for COVID RT-PCR tests?	Yes	No
9. If yes, how many COVID RT-PCR tests did you have? (Write the number of tests in the box opposite)		
10. Were you ever diagnosed with a COVID-19 infection?	Yes	No

**Please answer True or False to the following questions with a cross in the appropriate box (X).**

**Table:**

Knowledge regarding IPC for COVID-19	Place a cross over your chosen response	
1. The hospital environment (surfaces and water) is the main source of COVID-19 transmission to staff.	True	False
2. Handwashing with soap and water is sufficient to remove COVID-19 viral particles from the skin.	True	False
3. Alcohol hand rub is as good as handwashing with soap and water for decontaminating hands that are possibly contaminated with COVID-19.	True	False
4. Hand hygiene should be done before putting on a mask or N95 respirator.	True	False
5. Patients with COVID-19 infection require the same infection control precautions as patients infected with antibiotic-resistant bacteria.	True	False
6. Terminal cleaning and disinfection of the hospital room are required following the discharge of patients with COVID-19.	True	False
7. COVID-19 infection is spread mainly by respiratory droplets.	True	False
8. Open suctioning and intubations are aerosol-generating procedures.	True	False
9. The GSH Unit for Infection Prevention and Control performs active surveillance by testing every patient admitted to ICU for COVID-19.	True	False

**The attitude and perception of nurses on COVID-19 infection**

**Please answer the following questions with a cross in the appropriate box (X)**

**Table:**

Attitudes regarding IPC for COVID-19		Agree strongly 1	Agree 2	Neutral 3	Disagree 4	Disagree strongly 5
1.	I received adequate teaching about IPC during undergraduate and in-service training					
2.	I received sufficient training in IPC for COVID-19 before we began admitting infected patients to ICU					
3.	I received sufficient training in the procedures for safely donning and doffing PPE for COVID-19					
4.	GSH ICU have adequate isolation facilities to reduce potential infection transmission of COVID-19					
5.	Prevention of in-hospital COVID-19 staff and patient infections is the responsibility of the infection prevention and control (IPC) staff					
6.	Staff members who do not perform hand hygiene or ignore infection control recommendations should be reprimanded					
7.	The ICU nursing staff have a positive attitude towards COVID-19 infection prevention and control (IPC)					
8.	Transmission of COVID-19 infection can be prevented by taking antibiotics					
9.	The information and training about IPC for COVID-19 provided to ICU nursing staff were adequate to empower staff to protect themselves					
10.	The high workload during the COVID-19 pandemic resulted in decreased time to adhere to standard IPC practices in ICU					
11.	I am familiar with the COVID-19 IPC guidelines for ICU					
12.	During the COVID-19 pandemic, there has been adequate availability of PPE in ICU					
13.	During the COVID-19 pandemic, there has been adequate availability of cleaning supplies and disinfectants in the ICU					
14.	I consider working in the ICU to be a high risk for contracting COVID-19 infection					
15.	The correct PPE to care for COVID-19 patients is always available in the ICU					

**Practices regarding IPC for COVID-19**		**Agree strongly 1**	**Agree 2**	**Neutral 3**	**Disagree 4**	**Disagree strongly 5**

1.	I always wear the correct personal protective equipment (PPE) when dealing with COVID-19 infected patients					
2.	I always recognise and adhere to the transmission-based precaution signs in ICU					
3.	I have performed fit-testing for use of N95 respirators					
4.	When I am sick, I feel obliged to come to work because we are short-staffed					
5.	All staff in the ICU should receive an annual influenza vaccine					
6.	Staff who develop possible symptoms of COVID-19 should not report for duty					
7.	I perform hand hygiene before and after touching patients with COVID-19 infection					
8.	I perform hand hygiene after touching the patient’s surroundings like beds, tables, doors, etc					
